# Recent advances in the development of protein–protein interactions modulators: mechanisms and clinical trials

**DOI:** 10.1038/s41392-020-00315-3

**Published:** 2020-09-23

**Authors:** Haiying Lu, Qiaodan Zhou, Jun He, Zhongliang Jiang, Cheng Peng, Rongsheng Tong, Jianyou Shi

**Affiliations:** 1 Personalized Drug Therapy Key Laboratory of Sichuan Province, Department of Pharmacy, Sichuan Academy of Medical Science & Sichuan Provincial People’s Hospital, School of Medicine, University of Electronic Science and Technology of China, 610072 Chengdu, China; 2 Department of Ultrasonic, Sichuan Academy of Medical Science & Sichuan Provincial People’s Hospital, 610072 Chengdu, China; 3 Cancer Center, West China Hospital, Sichuan University and Collaborative Innovation Center for Biotherapy, 610041 Sichuan, China; 4 Miller School of Medicine, University of Miami, Miami, FL 33136 USA; 5 The Ministry of Education Key Laboratory of Standardization of Chinese Herbal Medicines of Ministry, State Key Laboratory Breeding Base of Systematic Research, Development and Utilization of Chinese Medicine Resources, Pharmacy College, Chengdu University of Traditional Chinese Medicine, 611137 Chengdu, China

**Keywords:** Target identification, Drug discovery, Target identification, Drug discovery, Target identification

## Abstract

Protein–protein interactions (PPIs) have pivotal roles in life processes. The studies showed that aberrant PPIs are associated with various diseases, including cancer, infectious diseases, and neurodegenerative diseases. Therefore, targeting PPIs is a direction in treating diseases and an essential strategy for the development of new drugs. In the past few decades, the modulation of PPIs has been recognized as one of the most challenging drug discovery tasks. In recent years, some PPIs modulators have entered clinical studies, some of which been approved for marketing, indicating that the modulators targeting PPIs have broad prospects. Here, we summarize the recent advances in PPIs modulators, including small molecules, peptides, and antibodies, hoping to provide some guidance to the design of novel drugs targeting PPIs in the future.

## Introduction

### PPIs and diseases

Proteins are the basic building blocks of life that are made by amino acids. The amino acids are coded by genes and form the peptides, peptides further form various proteins, and the proteins form the living tissues. Besides, proteins also have a central role in biological processes such as catalyze reactions, transport molecules, immune reactions to the various pathogens, and signal transduction between cells. What is more, the critical biological processes in the cells that directly associate with our health like DNA replication, transcription, translation, and transmembrane signal transduction all rely on the functional specific proteins. The aforementioned biological activities are regulated through protein complexes, which are typically controlled via protein–protein interactions (PPIs).^1–3^ PPIs in cells form a complicated network which has a term named “interactome”.^4,5^ The interactome has a significant role in physiological and pathological processes, including signal transduction, cell proliferation, growth, differentiation, and apoptosis, etc.^6–8^ Therefore, the aberrant PPIs are associated with many human diseases such as cancer, infectious diseases, and neurodegenerative diseases.^9–11^ Since the classic drug targets are usually enzymes, ion channels, or receptors, the PPIs indicate new potential therapeutic targets.^12^ In recent years, the PPIs have received increasing attention and became attractive targets.^13,14^ Recent studies indicate that the PPIs have great potential as an intervention target for novel treatment of refractory diseases, and its regulation is widely regarded as a promising strategy in drug discovery^8,15,16^ (Table 1).

**Table 1:** Summary of some PPI modulators in clinical trials

PPI	Related disease	Drug	Developer	Status	NCT number	Refs.
*Small molecules*						
MDM2/p53	Acute myeloid leukemia	Idasanutlin	Roche	Phase III	NCT02545283	^245^
MDM2/p53	Metastatic melanoma	AMG232	Amgen	Phase I/II	NCT02110355	^246^
MDM2/p53	Solid tumor with p53 wild type status	CGM097	Novartis	Phase I	NCT01760525	^247^
MDM2/p53	Advanced solid tumor, lymphoma	DS-3032b	Daiichi Sankyo	Phase I	NCT01877382	^248^
MDM2/p53	Neoplasm malignant	SAR405838	Sanofi	Phase I	NCT01636479	^249^
Bcl-2/Bax	Chronic lymphocytic leukemia	ABT-199	AbbVie	Approved in 2016	–	^250^
XIAP/caspase-9	Relapsed or refractory multiple myeloma	LCL-161	Novartis	Phase II	NCT01955434	^251^
XIAP/caspase-9	Recurrent head and neck squamous cell carcinoma	TL32711	National Cancer Institute	Phase I	NCT03803774	^252^
XIAP/caspase-9	Solid tumors, lymphoma	ASTX-660	Astex	Phase I/II	NCT02503423	^253^
XIAP/caspase-9	Solid cancers	GDC-0917	Genentech	Phase I	NCT01226277	^254^
PD-1/PD-L1	Prostatic neoplasms	CA-170	Astellas	Phase II	NCT01288911	^255^
Gp120/CCR5	HIV	Maraviroc	Pfizer	Approved in 2007	–	^256^
LFA-1/ICAM-1	Dry eye	Lifitegrast	Lifelong Vision Foundation	Phase IV	NCT03451396	^257^
Β-catenin/CBP	Liver cirrhosis	RPI-724	Komagome Hospital	Phase I/II	NCT03620474	^258^
Bromodomain/histone	Cardiovascular diseases	RVX-208	Resverlogix	Phase III	NCT02586155	^259^
Bromodomain/histone	NUT midline carcinoma	GSK525762	GSK	Phase I	NCT01587703	^260^
*Peptides*						
MDM2/p53	Advanced solid tumors, lymphomas	ALRN-6924	Aileron	Phase I/II	NCT02264613	^261^
*Antibodies*						
CD40/CD40L	Kidney transplantation	Bleselumab	Astellas	Phase II	NCT02921789	^262^
CD40/CD40L	Multiple myeloma	lucatumumab	Novartis	Phase I	NCT00231166	^263^
CD40/CD40L	Relapsed diffuse large B-cell lymphoma	dacetuzumab	Seattle Genetics	Phase II	NCT00435916	^264^
CD40/CD40L	Lupus Nephritis	BI655064	Boehringer Ingelheim	Phase II	NCT03385564	^265^
CD40/CD40L	Advanced solid tumors	ABBV-428	AbbVie	Phase I	NCT02955251	^266^
CD40/CD40L	Ulcerative colitis	ABBV-323	AbbVie	Phase II	NCT03695185	^267^
PD-1/PD-L1	Non-small lung cancer	Keytruda	Merck Sharp & Dohme	Approved in 2014	–	^268^
PD-1/PD-L1	Non-small lung cancer	Opdivo	Bristol Myers Squibb	Approved in 2014	–	^269^
PD-1/PD-L1	Non-small lung cancer	Tecentriq	Roche	Approved in 2016	–	^198^
PD-1/PD-L1	Merkel cell carcinoma	Bavencio	Merck and Pfizer	Approved in 2017	–	^270^
PD-1/PD-L1	Non-small lung cancer	Imfinzi	AstraZeneca	Approved in 2017	–	^199^
PD-1/PD-L1	Unresectable or metastatic melanoma	JS001	Shanghai Junshi Bioscience	Phase III	NCT03430297	^271^
PD-1/PD-L1	Advanced/metastatic solid malignancies	IBI308	Innovent Biologics	Phase I/II	NCT03568539	^272^
PD-1/PD-L1	Locally advanced or metastatic urothelial bladder cancer	BGB-A317	BeiGene	Phase II	NCT04004221	^273^

Data collected from https://clinicaltrials.gov [last accessed 7th June, 2020]

### Challenges in discovering PPIs modulators

The classic small molecule drug discovery approach mainly focuses on the protein–ligand interactions, such as enzymes, ion channels, or receptors, because these proteins typically contain a well-defined ligand-binding site that small molecules can interact with.^17^ The PPIs modulation through small molecules is generally considered difficult and PPIs were regarded as “undruggable” targets.^18,19^ It is estimated that there are about 130,000–650,000 types of PPIs in the human interactome.^4,8,20^ Although the number of protein complexes exceeds that of enzymes and receptors, designing a small molecule to bind to a PPI interface is challenging because of the reasons below. First, the PPIs occur on the interface of a specific domain where two identical or different proteins are in contact. The interface area of the interaction usually reaches 1500–3000 Å^2^,^21^ which is larger than that of receptor-ligand contact area (300–1000 Å^2^),^22^ and the interface is highly hydrophobic.^21^ Second, the PPIs interface tends to be flat and contains few grooves or pockets, thus making it difficult for the designed small molecule compounds to bind.^23–25^ Third, the amino acid residues involved in PPIs are either continuous or discontinuous in their respective protein structures, thus results in high-affinity binding between the proteins, making it difficult for the small molecular compounds to inhibit such high-affinity interaction.^26^ Forth, compared with traditional drug target enzymes or receptors, PPIs lack endogenous small molecular ligands for reference.^26^ Besides, compared to traditional small molecule drugs (200–500 Da), drugs acting on PPIs have a higher molecular weight (>400 Da), which makes it challenging to meet the criteria like Lipinski’s “rule of 5”.^23,27^

### Hot-spots

Theoretically, the large binding interfaces are not regarded as the ideal drug targets because it is difficult to find a matching molecule. However, the emergence of “hot-spots” makes the designing drugs for PPIs possible.^28^ Usually, PPIs happen on several amino acid residues in the interaction regions, having critical roles in the interaction. The regions of the amino acid residues on the PPIs interface that contribute to the binding-free energy are called “hot-spots”.^29–31^ As the area of PPIs expands, the number of hot-spots increases. The area of all hot-spots is about 600 Å^2^, usually located at or near the PPIs interface. The hot-spots in the PPIs are identified through a point mutation experiment. Specifically, the amino acid residues on PPI are muted into alanine, and the change of the binding-free energy is measured to determine the residues that contributes significantly to the binding-free energy. Hot-spots have been defined as these sites where alanine mutations cause a significant increase in the binding-free energy of at 2.0 kcal/mol.^32^ Tryptophan, arginine, and tyrosine are more likely to appear in hot-spots than other amino acids.^15,30^ Because of the important role of these “hot-spots” amino acids, they are often used to design PPI drugs. Therefore, although the interface of PPIs is relatively large, small molecule drugs only need to act on “hot-spots” to intervene in the PPIs.

### Current approaches for the discovery of PPI modulators

Targeting PPIs is challenging because of its unique interface. Compared to the binding pockets of conventional protein targets, the interface of PPIs tends to be flat. Therefore, classic medicinal chemistry methods are less effective for designing and identifying PPIs modulators. Thus, it is necessary to develop more effective approaches for screening the PPI modulators. A wide variety of strategies have been developed to identify hits and leads of PPI modulators in recent years.

#### High-throughput screening

High-throughput screening (HTS) is a well-established method for discovering classic drug targets. It has been used to identify compounds that target the hot-spots of PPI interfaces.^16^ Because of the particularity of PPI interface, the compound library used for screening conventional targets may not be suitable for screening PPI modulators. It’s crucial to have a broad compound library to have chemical diversity that may match the PPI target. However, HTS has been proved to be useful in the identification of molecules at the initial stage. For example, it successfully screened out inhibitors against MDM2/p53 interaction.^33–35^

#### Fragment-based drug discovery

Fragment-based drug discovery (FBDD) aims to identify molecular fragments from fragment libraries.^36^ Compared to HTS, FBDD is a better approach for PPIs modulators designing because the PPI interface often consists of discontinuous hot-spots. Surface plasmon resonance (SPR), nuclear magnetic resonance (NMR), X-ray crystallography, and mass spectroscopy (MS) can be utilized for discovery and validation of the fragment hits.^37,38^ Once the fragment hits are identified, the fragment linking, fragment optimization, and fragment self-assembly can be used to obtain the hits.^39^ Because the molecular weight of fragments is low and the contact interface is limited, the affinity is relatively low.^40^ The X-ray crystallography and NMR can provide structural information for the hits optimization. As a result, FBDD is not suitable for the targets with unknown structure. The examples of successful application of FBDD in PPI modulators’ discovery include XIAP/caspase-9,^41^ Bcl-2/Bax,^42^ and bromodomains,^43^ etc.

#### Structure-based design

Since most PPIs lack endogenous small molecule ligands, it is challenging to rationally design the associate PPI modulators. However, the hot-spots provide important structural information and a basis for the rational design of PPI modulators. At present, there are two design strategies for structure-based design PPI modulators. The first is based on the hot-spots structure. Through bioisosterism and de novo design, the novel small molecule modulators can be obtained.^44^ For example, during the development of VHL/HIF1α PPI inhibitors, Hyp564 was identified as a crucial amino acid. Through the de novo design targeting the Hyp546, the inhibitors were obtained.^45,46^ The second is peptidomimetic design which mainly rely on computer modeling and phage display to simulate the secondary structure of the key peptides in PPIs. Furthermore, small molecules were designed or binding peptides were synthesized based on the stable α-helix structure formed by the key peptides.^47^ The α-helix is the most common identified secondary structure in PPIs.^48^ At present, many PPI modulators have been successfully developed based on the α-helix structure, including c-Myc/Max,^49^ Bcl-2/Bax,^50^ and MDM2/p53.^51^

#### Virtual screening

The virtual screening is based on professional application software to screen out hits from compound libraries. One big challenge in developing PPI modulators is to identify the disease-related and druggable PPIs among thousand of available ones. The virtual screening may be useful to locate the binding sites by analyzing the protein surface. It can be classified into both a structure-based approach and a ligand-based approach. The ligand-based approach aims to screen compounds that satisfy the built pharmacophore model. In contrast, the structure-based approach relies on the structural information of the target protein. The virtual screening was successfully applied in the development of PPI modulators including Ubc13/Uevl,^52^ MDM2/p53,^53^ and TCF/β-catenin.^54^

### Mechanism of PPIs modulators

**Fig. 1:**
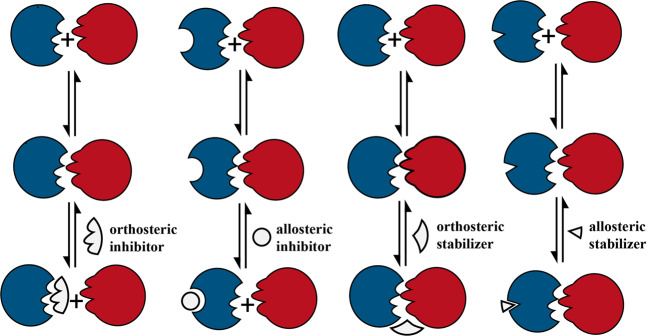
Orthosteric and allosteric mechanisms for PPI inhibition and stabilization

The small molecule PPIs modulators can interact not only with protein–protein interface but also with allosteric sites^55,56^ (Fig. 1). Studies showed the small molecule modulators can either bind to the non-interaction region of the proteins which is named allosteric inhibition or bind to the PPI interface, which is named orthosteric inhibition. Besides PPI inhibition, some modulators can stabilize or even enhance PPI. There are two models to explain the stabling effects: when the modulator binds to the allosteric regulatory site of the protein, it triggers the conformation change of the target protein, thereby enhance the affinity of the target protein to the other protein. In case the modulator binds to the PPI interface, provides more contact sites for the two proteins, the binding force of the two proteins gets enhanced.^57^ For the PPI with hot-spots, the corresponding ligands can be designed to directly affect PPI. In case the PPI without hot-spots, the PPI can be indirectly regulated through the allosteric mode.^58^ Specifically, if the PPI hot-spots residues gather together and form appropriate pockets, the orthosteric modulators can be designed and developed based on the pockets structure information to directly influence the associate PPI. If the hot-spots can not form appropriate binding sites, developing the allosteric modulators will be a better choice.^59^ Most of the small molecules that have been identified to modulate PPIs are inhibitors. The PPI stabilization represents a promising modulation approach since the combination with pre-existing complexes is more advantageous in energy saving compared to the inhibition of complexes formation.^60–62^ However, the development of PPIs stabilizers has not received sufficient attention as compared to the development of PPIs inhibitors.^63^

### Three types of PPIs modulators

Up to date, the PPI modulators can be classified into three categories (Table 2). The first category is the small molecule modulators. Compare to the classic drug targets like enzymes or ion channels, the PPI interface is large, flat, and lacks a suitable size pocket which the small molecules can bind with. What is more, the PPI interface is usually hydrophobic. Therefore, a potent PPI modulator should cover a large surface area and make a large number of hydrophobic contacts. Such a modulator may face pharmacokinetic issues due to its large molecular weight and poor solubility.^8^ Therefore, the small molecule modulator is more suitable for the tight and narrow PPI interface.^44^ The second category is an antibody. When targeting a large PPI interface, an alternative other than small molecular compounds is needed to cover the large interface. Although monoclonal antibodies compete with PPIs, because of their large molecular weight, the application of monoclonal antibodies is limited to the extracellular targets. Up to date, the monoclonal antibodies have been successfully used in clinical treatment although they may trigger adverse reactions associated with the immune reactions. The third category is peptides. The peptides are designed based on the structure information of the hot-spots.^64^ The designed peptides retain the key roles when they bind to proteins, thereby forming a strong affinity with the proteins. Compared with small-molecule PPI modulators and monoclonal antibodies, the molecular weight of peptide is between the two. It has higher target specificity and affinity and is a potential PPI modulator. However, the peptide is susceptible to hydrolysis by various hydrolases in the body, which makes its half-life short.

**Table 2:** The advantages and disadvantages of three types of PPI modulators

Small molecules	Peptides	Antibodies
*Advantages*		
Penetrate cell membrane Oral administration	Target specificity High affinity	Strong target specificity High efficiency
*Disadvantages*		
Side effects (low selectivity)	Short half-life Poor oral administration Unstable physicochemical properties Poor solubility	Side effects (immunogenicity) Generally act on extracellular targets (huge molecular weight)

In this review, we summarized the latest advances in PPIs modulators development including the small molecules, peptides, and antibodies. Also, we summarized the up to date some PPIs modulators in clinical trials, hoping to provide some guidance to the design of novel drugs targeting PPIs in the future.

## Inhibitors of PPIS

### Inhibitors of MDM2/p53 interaction (small molecules, peptides)

**Fig. 2:**
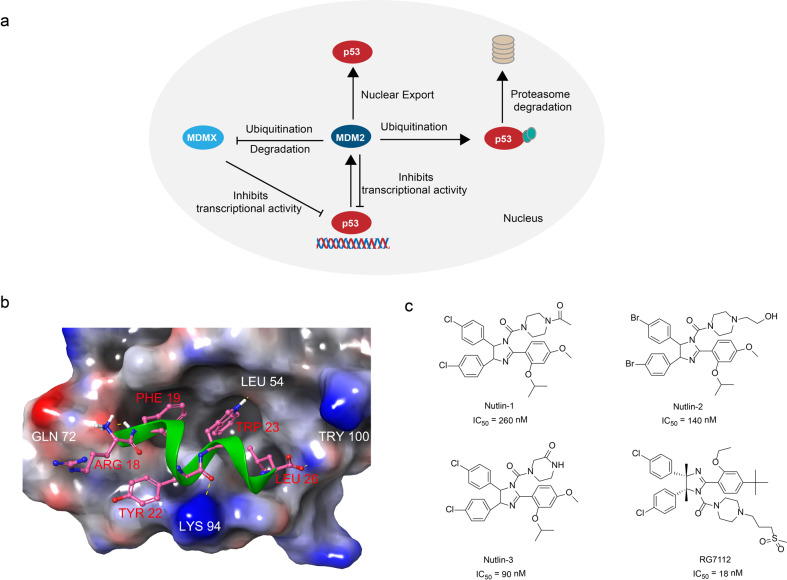
The p53/MDM2 interactions and inhibitors. **a** The p53/MDM2 signaling pathway: MDM2 directly binds to p53 and inhibits its transcriptional activity, causes ubiquitination and proteasomal degradation of p53, and exports p53 out of the nucleus which promotes p53 degradation. **b** MDM2 (surface)-p53 peptide (green) complex (PDB:1T4F). **c** The chemical structures of inhibitors of MDM2/p53

The p53 is an important protein that regulates the cell cycle and functions as a tumor suppressor.^65^ Studies showed ~50% human cancers have alterations in the p53 gene which results in the inactivation of p53 function or loss of p53 expression.^66^ The mouse double minute 2 (MDM2) is a proto-oncogene and a key negative regulator of p53. A negative feedback loop between MDM2 and p53 has been uncovered as the mechanism of how they regulate each other’s level in the cells (Fig. 2a).^67^ MDM2 directly binds to and forms a complex with p53, inhibiting the transactivation of p53. Therefore, recovering the impaired the function of p53 by disrupting the MDM2/p53 interaction offers a potential approach for the treatment of cancer.^68,69^

The X-ray crystallography disclosed the details of the MDM2/p53 interaction. The interaction between MDM2 and p53 involves four key hydrophobic residues (Phe19, Leu22, Trp23, Leu26) in an α-helix formed by p53 and a small but deep hydrophobic pocket in MDM2^28^ (Fig. 2b). An effective strategy to block their interaction is to design a small molecule compound that mimics the “hot-spots” residue structure of p53, which competes with p53 to bind with MDM2, thereby preventing the inactivation of p53. The peptide-like design, HTS, and structure-based design were adopted as the strategies to screen the MDM2/p53 inhibitors with good drug-like properties.^70–72^

The imidazoline compounds Nutlins discovered by Vassilev et al.^33^ through HTS showed strong inhibitory effects against MDM2/p53 interaction (Fig. 2c). As a group of small-molecule inhibitors of MDM2, the Nutlins mimic the effect of p53 peptide segment. The Nutlins bind to the deep hydrophobic pocket in MDM2, therefore block the MDM2/p53 interaction. Studies showed the IC_50_ of Nutlin-1, Nutlin-2, and Nutlin-3 on MDM2/p53 interaction were 260, 140, and 90 nM, respectively, in vitro.^33^ Based on the inhibitory dose values, the Nutlin-3 was selected as the lead compound. Roche restructured the Nutlin-3 by substituting the methyl for the 4- and 5-position hydrogen atoms of its imidazole ring, and replaced the cyclomethoxy group at the para position of the benzene ring with a *tert*-butyl group which prevented the metabolic inactivation of the imidazole ring and the benzene ring.^73^ Meanwhile, the isopropoxy group was replaced by an ethoxy group to reduce the molecular weight, and the hydrophilic side chain of carbonyl piperazine was replaced by a methylsulfonyl propyl piperazine, therefore obtained the compound RG7112 (Fig. 2c). The homogeneous time-resolved fluorescence (HTRF) assay showed that the compound RG7112 (IC_50_ = 18 nM) was optimized to be four times more sensitive than that of Nutlin-3.^73^ RG7112 is the first MDM2 inhibitor entered clinical trials for the treatment of advanced solid tumors.

Using peptides to inhibit PPIs has become a promising way to discover active compounds. Chang et al.^74^ reported a class of potent MDM2 peptide inhibitors ATSP (Table 3), among which the IC_50_ value of ATSP-7041 reached 0.9 nM, and the reported *K*_*i*_ values of the ATSP series peptides reached the nanomolar level. The key is that the peptide mimics the key α-helical structure in the p53/MDM2 interaction, thus binding MDM2 competitively with p53. ATSP inhibitors showed a certain biological activity in vivo, which may be related to the good cell membrane permeability produced by the stable α-helix structure. The western blot analysis also showed that the ATSP inhibitors inhibit MDM2 in cells, thereby activating the role of tumor suppressor protein p53.^74^

**Table 3:** Peptide inhibitors of MDM2/p53 interaction reported by Chang et al.

Name	Sequence	*K*_*i*_ (nM)
ATSP-1800	Ac-Gln-Ser-Gln-Gln-Thr-Phe-R8-Asn-Leu-Trp-Arg-Leu- Leu-S5-Gln-Asn-NH2	25.9
ATSP-3848	Ac-Leu-Thr-Phe-Glu-His-Tyr-Trp-Ala-Gln-Leu-Thr-Ser-NH2	14.6
ATSP-3900	Ac-Leu-Thr-Phe-R8-His-Tyr-Trp-Ala-Gln-Leu-S5- Ser-NH2	1.0
ATSP-4641	Ac-Leu-Thr-Phe-R8-Ala-Tyr-Trp-Ala-Gln-Leu-S5- Ser-NH2	4.9
ATSP-6935	Ac-Leu-Thr-Phe-R8-Glu-Tyr-Trp-Ala-Gln-Leu-S5- Ser-NH2	1.2
ATSP-7041	Ac-Leu-Thr-Phe-R8-Glu-Tyr-Trp-Ala-Gln-Cba-S5- Ser-Ala-Ala-NH2	0.9
ATSP-7342	Ac-Leu-Thr-Ala-R8-Glu-Tyr-Trp-Ala-Gln-Cba-S5- Ser-Ala-Ala-NH2	536

### Inhibitors of Bcl-2/Bax interaction (small molecules)

**Fig. 3:**
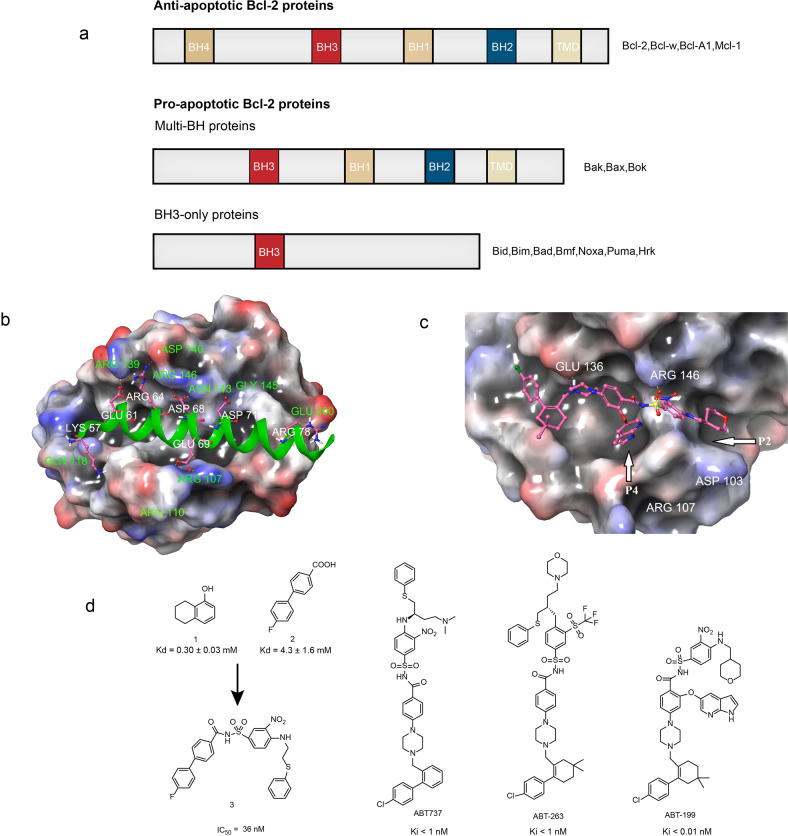
The Bcl-2/Bax interactions and inhibitors. **a** The Bcl-2 family can be classified into two categories: the anti-apoptosis proteins and pro-apoptosis proteins. The pro-apoptosis proteins can be divided into multi-BH proteins and BH3-only proteins. **b** The crystal structure of Bcl-2 in complex with Bax BH3 peptide (PDB:2XA0). **c** The binding modes of ABT-199 binds to Bcl-2 (PDB:6GL8). **d** The chemical structures of inhibitors of Bcl-2/Bax

The Bcl-2 family is a key regulator of apoptosis, and it has over twenty members. According to their role in apoptosis, the Bcl-2 family members can be divided into two categories including the anti-apoptotic proteins and the pro-apoptotic proteins (Fig. 3a). The anti-apoptotic proteins include Bcl-2, Bcl-w, Mcl-1, and Bcl-A1. The pro-apoptotic proteins include Bax, Bok and Bak, Bid, Bad, Bmf, Noxa, Puma, Hrk (among them, Bid, Bad, Bmf, Noxa, Puma, and Hrk are BH3-only protein).^75,76^ Both anti-apoptotic and pro-apoptotic members usually synergize in the form of dimers, having the role of apoptotic switch.^77,78^ Pro-apoptotic proteins such as Bax and Bad have critical roles in the apoptosis. The functions of these pro-apoptotic proteins are blocked when they bind to the anti-apoptotic proteins like Bcl-2. Therefore, inhibiting the interaction between the pro- and anti-apoptotic proteins prevents the tumor cells from escaping apoptosis.

The Bcl-2 family members have low homology, but they contain at least one or four conserved Bcl-2 homology (BH) motifs, named BH1, BH2, BH3, and BH4.^76^ There are two hydrophobic ɑ-helix structures in Bcl-2 which are surrounded by six to seven amphiphilic ɑ-helix structures, of which four amphiphilic ɑ-helix structures form a hydrophobic BH3 “pocket” to interact with Bax (Fig. 3b).^79^ Compared with the Bax/Bak homodimer, the Bcl-2/Bax homodimer is more stable, which weakens the role of Bax/Bak in inducing cell apoptosis and prevents cell apoptosis. Therefore, the lead compounds should mimic the function of the pro-apoptotic protein domain. The ideal compounds will bind to the hydrophobic pocket on the surface of the anti-apoptotic protein, thereby blocking the anti-apoptotic protein to bind with the BH3 domain and result in the cancer cell apoptosis induction.^80,81^

Abbott researchers studied the Bcl-XL hydrophobic groove and found that the hydrophobic groove consists of two relatively independent small pockets.^82^ They used the “SAR by NMR” approach to screen the fragments with BH3 on Bcl-XL, and obtained compound 1 (*K*_d_ = 0.30 ± 0.03 mM) and compound 2 (*K*_d_ = 4.3 ± 1.6 mM) from the library (Fig. 3d). The researchers used a fragment-based drug design strategy and screened the compounds based on the NMR data. Based on the position and spatial orientation data obtained from the complexes of the Bcl-XL hydrophobic groove-binding pockets with the compound 1 and compound 2, the researchers modified the compound 2’s structure by adding a linking group thereby constructed a highly active new lead compound 3 (IC_50_ = 36 nM). However, the compound 3 exhibited poor water solubility but high affinity to human serum albumin (HSA). In subsequent structural optimization, the researchers reduced the compound’s affinity to HAS through substituting polar groups at specific sites. It was found that introducing 2-dimethylaminoethyl substituent at the second ligand of compound 3 and substituting piperazine at the first ligand improved its affinity to Bcl-2 protein, and thus compound ABT-737 was obtained (Fig. 3d). The ABT-737 binds to Bcl-XL (*K*_*i*_ < 0.5 nM) and Bcl-2 (*K*_*i*_ < 1 nM), and its IC_50_ value reaches 35 nM in 10% of human serum. The ABT-737 is not only widely used in the biological studies associated with apoptosis, but also in preclinical studies in lymphoma, small cell lung cancer and chronic lymphoblastic leukemia.^83,84^

However, the poor oral absorption of ABT-737 significantly limits its clinical application. The ABT-263 (Navitoclax) (Fig. 3d) is the second generation of Bcl-2 anti-apoptotic protein inhibitor based on the structure of ABT-737.^85,86^ It can bind with Bcl-2 (*K*_*i*_ < 1 nM), Bcl-XL (*K*_*i*_ < 0.5 nM), Bcl-w (*K*_*i*_ < 1 nM), and MCL-1 (*K*_*i*_ = 550 nM). The preclinical studies showed that ABT-263 alone effectively inhibited the small cell lung cancer xenograft tumors growth in mice model. Besides, the ABT-263 also showed synergic effects in inhibiting the solid tumors and blood tumors in combination with other antineoplastic agents.^86^ However, studies also showed the ABT-263 could temporarily decrease the platelet count.^87^

The ABT-199 (Venetoclax) (Fig. 3d) is the first small molecule PPI inhibitor approved for marketing. It is a Bcl-2 selective inhibitor based on the structure design of lead compound ABT-263.^88^ It was approved to be marketed in 2016 for the treatment of chronic lymphoblastic leukemia.^89^ By studying the complex structure of Bcl-2 protein and small molecule acyl sulfonamide compounds, it was found that the introduction of indole group was beneficial to enhance the binding of drugs to P4 pockets through hydrophobic interaction and resulted in the formation of electrostatic interaction with aspartic acid residues specific to Bcl-2 protein^88^ (Fig. 3c). The researchers from the Abbvie introduced indole group and azaindole group into the ABT-263 skeleton structure and studied the structure–activity relationship. The studies showed that the ABT-199 had good activity on Bcl-2-dependent hematological cancers.^88^ The ABT-199 showed a high affinity for Bcl-2 (*K*_*i*_ < 0.01 nM) and a weak affinity for Bcl-XL (*K*_*i*_ = 48 nM). It showed an excellent inhibitory effect on the acute lymphoblastic leukemia cells with high expression of Bcl-2 (EC_50_ = 8 nM). Compared with the second-generation drug ABT-263, the ABT-199 significantly reduced the damage to the platelets in both in vitro and in vivo studies.

### Inhibitors of XIAP/caspase-9 interaction (small molecules)

**Fig. 4:**
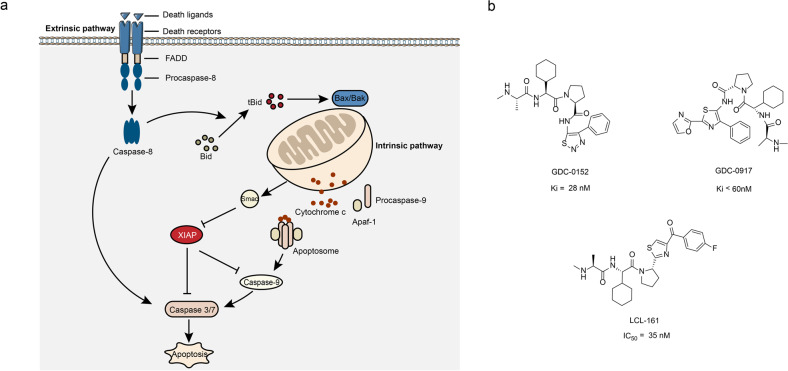
The XIAP/caspase-9 interactions and inhibitors. **a** The apoptotic pathway. There are two apoptotic pathways: extrinsic and intrinsic. The extrinsic pathway (also known as death receptor) involves the binding of a death receptor ligand to a member of the death receptor family. Active caspase-8 cleaves and activates the executioner caspase-3 and caspase-7, leading to the cell death. The intrinsic pathway (also known as mitochondrial) is mediated by caspase-9. After the mitochondrial membrane is stimulated by apoptosis, it releases cytochrome c and Smac proteins into the cytoplasm. Smac is a pro-apoptotic protein. Cytochrome *c* combines with Apaf-1 to form a polymer, and promotes pro-caspase-9 to form apoptotic bodies, and then activate caspase-9. The activated caspase-9 can activate other caspases, such as caspase-3, so as to induce apoptosis. **b** The chemical structures of inhibitors of XIAP/caspas-9

Inhibitors of apoptosis proteins (IAPs) are an important class of endogenous anti-apoptotic proteins.^90^ They bind to the caspase or other pro-apoptotic proteins, results in the inhibition of the pro-apoptotic proteins functions and promotes their degradation, thereby regulates the apoptosis.^91,92^ The IAPs has eight family members: XIAP, c-IAP1, c-IAP2, ML-IAP/Livin, ILP2, NAIP, Bruce/Apollon, and surviving.^93^ The caspase, a cysteine-containing aspartate proteolytic enzyme, is the main implementer of apoptosis, which induces apoptosis through two pathways. One of which is the death receptor pathway (extrinsic pathway) that mediated through caspase-8. The other one is the mitochondrial pathway (intrinsic pathway), which mediated via cytochrome C/caspase-9 (Fig. 4a).^94^ The BIR3 domain of the XIAP binds to and inhibits pro-apoptotic caspase-9, thus suspends the apoptosis.^95^ Interestingly, the endogenous protein inhibitor of the XIAP–caspase-9 interaction exists in the form of Smac (second mitochondria-derived activator of caspase). When the Smac released from the mitochondria, its N-terminal amino acids, alanine–valine–proline–isoleucine (AVPI) bind to the BIR3 domain of XIAP, which makes the XIAP lose the ability to combine with caspase, so as to promote apoptosis.^96,97^

The four amino groups of the AVPI at the N terminus of the Smac protein have a very important role in the binding of XIAP to caspase-9, which competes with the caspase-9 protein for binding to XIAP.^98,99^ Therefore, the interaction between XIAP and caspase-9 can be inhibited by the Smac protein mimetics that exhibit the similar affinity to XIAP.^100^ The crystal structure of Smac and XIAP-BIR3 domain revealed that the Val of P2 position and the Ile of P4 position in the Smac formed three hydrogen bonds with the Gly306 and the Thr308 of XIAP-BIR3 domain.^99^ The 3-position Pro ring bind with the hydrophobic region formed by the Trp323 and Tyr324 of XIAP-BIR3 domain through van der Waals force. Moreover, the Pro ring is essential for maintaining the conformation of AVPI peptide chain, so proline is relatively stable and usually not replaced by other amino acids. Flygare et al.^101^ discovered the first Smac simulator GDC-0152 through a combination of peptide-like design strategies and high-throughput screening (Fig. 4b). The GDC-0152 binds to XIAP-BIR domain with high affinity by mimicking the structure of the Smac AVPI peptide. Another Smac mimetic GDC-0917 (CUDC-427) (Fig. 4b) has entered phase І clinical trials for the safety evaluation of patients with advanced solid tumors and lymphomas.^102^ Novartis LCL-161 (Fig. 4b), which is currently progressing rapidly, has entered phase II clinical trials for triple negative breast cancer.^103^

### Inhibitors of Hsp90/Cdc37 interaction (small molecules)

**Fig. 5:**
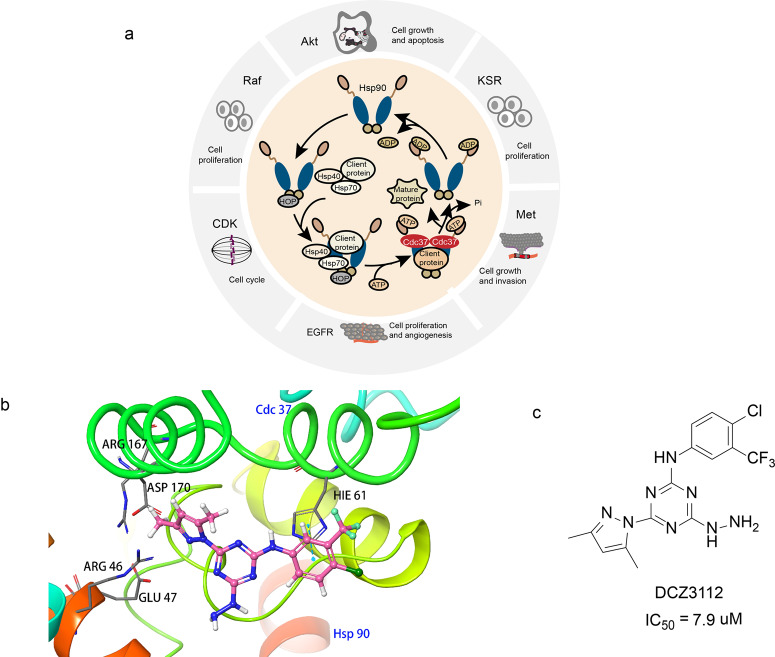
The Hsp90/Cdc37 interactions and inhibitors. **a** Co-chaperone regulation of client protein activation. In the chaperone cycle of Hsp90, the open state Hsp90 firstly combined with HOP through its C terminal. Subsequently, it recruits Hsp40, Hsp70, client protein, and Cdc37 to form a mature complex. After ATP hydrolysis, ADP and mature client protein are released. Hsp90 is converted into an open state and enters the next ATP cycle. **b** The complex structure DCZ3112 and the N-terminal domain of Hsp90 modeled by molecular docking based on the crystal structure of Hsp90–Cdc37 complex (PDB:2K5B). **c** The chemical structures of inhibitors of Hsp90/Cdc37

The heat shock protein 90 (Hsp90) is a widely existed, highly conserved molecular chaperone that was discovered in 1962. It is also one of the most abundant proteins in cells. Studies showed the expression of Hsp90 in tumor cells is two to ten times higher than that of normal cells, which indicates it has a very important role in tumor cell growth and survival.^104^ Studies also showed the Hsp90 participates in the maturation of protein kinases and transcription factors such as Her2, VEGF, mutant p53, CDK4, HIF-1ɑ, Raf -1, Akt, etc. which regulate the cancer cell’s growth and apoptosis signaling pathways.^105,106^ Hsp90 stabilizes the conformation of the client proteins mentioned above and prevents them from ubiquitination-mediated degradation, thereby stabilizes them to stay in the active form and promotes the tumor growth and metastasis (Fig. 5a).^107^ Therefore, inhibiting the interaction between the Hsp90 and its client proteins may promote the degradation of the client proteins, and thus results in the inhibition of the tumor growth.^108–110^

The previous studies also showed that the Hsp90 is a homodimer and each monomer is formed by three highly conserved domains including a N-terminal ATP-binding domain, a middle domain, and a C-terminal dimerization domain.^111^ The N-terminal domain is an ATP/ADP-binding site that hydrolyzes the ATP in the ATP-binding pocket. The ATP/ADP-binding site acts as a conformational transformation region, regulates the assembly of the Hsp90 involved multi-molecular chaperone complexes.^112^ The middle domains serve as both nuclear localization sequences and the target protein-binding sites. The middle domains distinguish the different substrate proteins and regulate the activity of specific substrates of molecular chaperones.^113^ The C-terminal domain is a self-dimerization site of Hsp90, which enhances the interaction of two Hsp90 N-terminal domains.^114^ The current Hsp90 inhibitors can be classified into three categories: the N-terminal ATP pocket inhibitors, the C-terminal nucleotide site inhibitors, the Hsp90 and chaperone complexes inhibitors. One critical function of Hsp90 is to regulate its client proteins to utilize ATP. The inhibition of such crucial function affects many normal proteins, results in high toxicity.^115^ Hsp90 inhibitor SNX-5422 developed by Pfizer terminated clinical phase 1 trial in 2011 due to ocular toxicity.^116^ Therefore, researchers believe that targeting Hsp90 and its molecular chaperones is a new direction for the cancer treatment study.^117,118^

Among the numerous molecular chaperones of Hsp90, the value of cell division cycle protein 37 (Cdc37) has attracted much attention.^110^ Many protein kinases (such as EGFR, CDK, Akt) rely on Cdc37 to aggregate onto Hsp90, thus completing the correct folding of the complex’s spatial conformation.^110,119^ Therefore, inhibiting the interaction of Hsp90 and Cdc37 may deactivates the kinase client proteins, thereby inhibiting the proliferation and growth of tumor cells. In addition, the PPI targeting Hsp90/Cdc37 specifically targets the kinase client protein of Hsp90, thereby improving selectivity and avoiding a series of adverse reactions.

In 2004, the researchers resolved the first crystal structure of Hsp90_N_-Cdc37_M_ which provided a solid structural basis for the design of Hsp90/Cdc37 interaction inhibitors.^120^ The NMR analysis of the Hsp90_N_–Cdc37_M_ complex indicated the hydrophobic interaction is the major interaction force between the two proteins.^121^ The key amino acids of the interface include Met164, Trp193, Ala204, Leu205 of Cdc37_M_ and Ala117, Ala121, Ala124, Ala126, Met130, Phe134 of Hsp90_N_. The Leu205, the leucine residue of Cdc37, is very important for the formation of Hsp90_N_–Cdc37_M_ complex. Experiments show that the mutation of Leu205 resulted in the loss or decrease of the binding ability between Hsp90_N_ and Cdc37_M_.

In 2018, Xie et al.^107^ first reported the small molecule inhibitor DCZ3112 inhibits the interaction of Hsp90/Cdc37 (Fig. 5c). The DCZ3112 directly binds to the N-terminal domain of Hsp90, inhibits the Hsp90/Cdc37 interaction without affecting the ATPase activity of Hsp90 (Fig. 5b). The DCZ3112 mainly inhibits the proliferation of HER2-positive breast cancer cells and its IC_50_ value of SK-BR-3 and BT-474 cells was 7.9 and 4.6 μM, respectively. Experiments in SK-BR-3 and BT-474 cells showed that DCZ3112 downregulated the number of Hsp90 client proteins HER2, Akt, RAF-1, CDK4, and CDK6 in a concentration-dependent manner. The in vitro experiments results showed the DCZ3112 has a synergistic effect in inhibiting cell proliferation, inducing G1 arrest, inducing apoptosis, and reducing phosphorylation of Akt and Erk.^107^

### Inhibitors of c-Myc/Max interaction (small molecules)

The c-Myc is a transcription regulator encoded by the proto-oncogene Myc. It is a highly conserved protein with helix structure. It has a critical role in promoting tumorigenesis, maintaining the growth, proliferation and differentiation of tumor cells, angiogenesis and apoptosis.^122–124^ Aberrant expression of c-Myc has been confirmed in most malignant tumors.^125^ As a result, c-Myc has become a research hot spot. The c-Myc has a bHLH-ZIP domain, its function depends on the formation of Myc–Max dimer.^126^ The Myc–Max dimer recognize the CACGTG in E-box sequence on their target DNA and bind to it to activate or enhance the transcription of the regulated genes.^126^ Therefore, inhibiting the PPI between the c-Myc and Max may inhibit the activation or transcription of oncogenes, indicating an antitumor effect.^127,128^

**Fig. 6:**
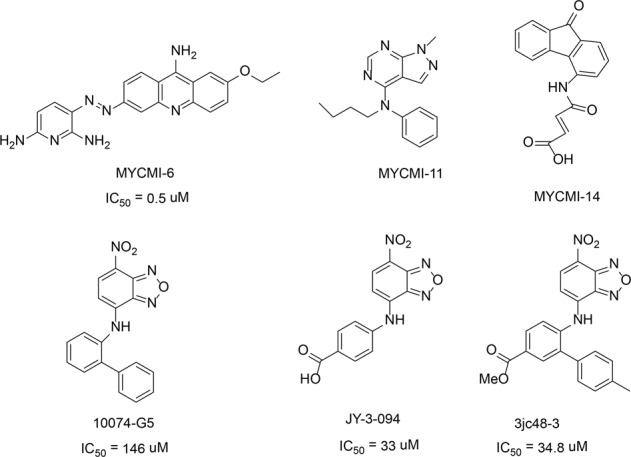
The chemical structures of inhibitors of c-Myc/Max

Castell et al.^129^ used the cell-based bimolecular fluorescence complementation (BiFC) to screen small molecules that interfere with c-Myc/Max interaction. Three compounds with good potential were identified from a library of 1990 compounds: MYCMI-6, MYCMI-11 and MYCMI-14 (Fig. 6). MYCMI-6 showed a strong inhibitory effect on c-Myc/Max interaction in both in vitro and cell-based experiments. The surface plasmon resonance (SPR) results showed that MYCMI-6 blocks the c-Myc-driven transcription and MYCMI-6 selectively binds to the bHLH-ZIP domain of c-Myc (*K*_d_ = 1.6 ± 0.5 μM). The above results indicate that the MYCMI-6 inhibits the growth of cancer cells in a c-Myc-dependent manner (IC_50_ = 0.5 μM) and has no effect on normal cells. Also, studies showed MYCMI-6 induces apoptosis, inhibits the proliferation of tumor cells and reduce the microvessel density in the mice xenograft tumors. The validation experiments based on microscale thermophoresis (MST) and SPR showed that MYCMI-6 binds to the bHLH-ZIP domain of c-Myc (*K*_d_ = 4.3 μM) and Max (*K*_d_ = 3.8 μM), respectively, thus inhibit the interaction between c-Myc/Max proteins.

Chauhan et al.^130^ discovery that the compound 10074-G5 inhibits the formation of heterodimers between c-Myc/Max (Fig. 6). The nitro and furan rings of 10074-G5 interact with Arg366, Arg367, and Arg372 in the HLH domain, therefore, inhibit the formation of heterodimers between c-Myc and Max.^128^ The optimization of compound 10074-G5 led to the discovery of compound JY-3-094 (Fig. 6).^131^ The electrophoretic mobility shift assays (EMSAs) showed the formation of heterodimers between JY-3-094 and c-Myc/Max was five times more active than the 10074-G5 (IC_50_ = 33 μM vs 146 μM). However, unlike 10074-G5, the JY-3-094 does not inhibit the proliferation of human promyelocytic leukemia (HL60) or Daudi Burkitt lymphoma cell lines because the charged carboxylic acids groups in the molecule impeded the cell entry. By esterifying the carboxylic acid in the JY-3-094 into a series of ester prodrugs, the lower IC_50_ values were reached in both HL60 and Daudi Burkitt lymphoma cell lines. However, the activity of ester prodrugs is always limited by the activity of carboxylic acid metabolites, so the structural optimization of JY-3-094 continuous. Studies showed the phenyl ring adjacent to aniline in 10074-G5 enhanced the inhibitory effects. The introduction of phenyl ring into the JY-3-094 led to the formation of 3JC48-3, with an IC_50_ value of 34.8 μM for c-Myc/Max protein inhibitory activity (Fig. 6). Further studies showed that 3JC48-3 inhibits the tumor cell proliferation by inducing cell arrest in the G0/G1 phase. Such a significant increase of c-Myc/Max protein inhibitory effect may be the interaction between phenyl ring with the Phe375/lle381 and Arg378 in the c-Myc/Max.^130^

### Inhibitors of KRAS/PDEδ interaction (small molecules)

**Fig. 7:**
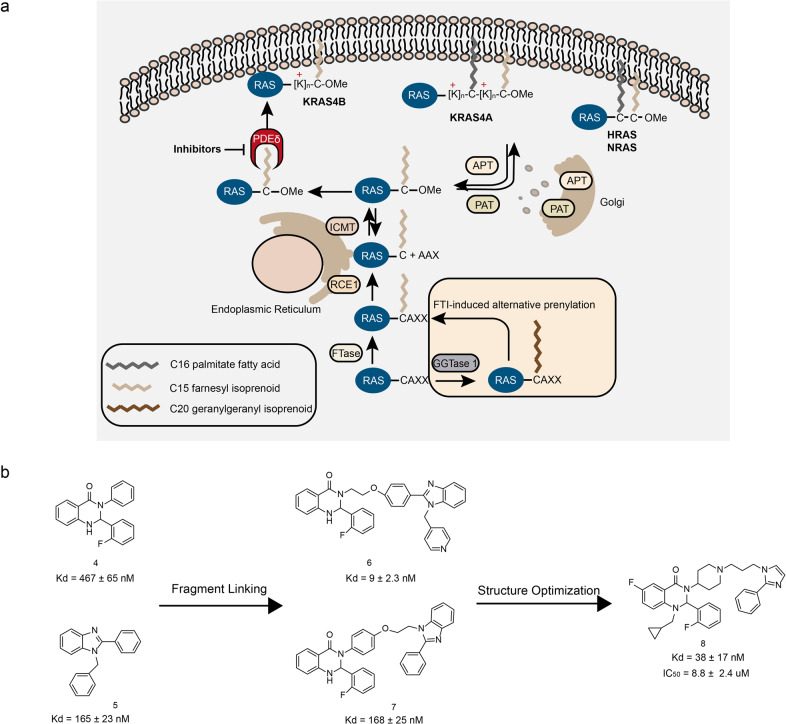
The KRAS/PDEδ interactions and inhibitors. **a** The process of KRAS localization to the plasma membrane. **b** The chemical structures of inhibitors of KRAS/PDEδ

Oncogenic RAS is an important antitumor target, and are mutated in about 20–30% of human cancers.^132^ The RAS family has three members: HRAS, KRAS, and NRAS. The KRAS protein is often mutated in various cancers. Specially, the KRAS mutation has been observed in a large proportion of pancreatic cancers.^133^ The RAS mutations lead the cells on the hyperactive state for unlimited proliferation. As a molecular switcher, RAS activates the downstream signaling pathways such as MAPK and PI3K-Akt through binding to GTP, thus regulating the growth, proliferation, differentiation, and apoptosis of the cells. If the RAS proteins are continuously activated, it can bind to the downstream effector proteins and transmit signals to the downstream proteins, causing aberrant cell proliferation or tumorigenesis.^134^ Therefore, RAS proteins can be developed as an important target for the cancer treatment. At present, there are mainly two strategies to inhibiting KRAS. The first strategy is directly targeting the signal pathway of KRAS protein. The second strategy is inhibiting the KRAS membrane association which impairs the localization of KRAS and the signal transduction of tumor proliferation. To carry out its signal transduction function, the RAS proteins need to be recruited to the inner side of the plasma membrane after expression.^135^ In the process of KRAS relocating to the cell membrane, PDEδ promotes the KRAS protein recruit to the Golgi apparatus^135,136^ (Fig. 7a). Therefore, through interfering with the interaction between PDEδ and KRAS, the localization of KRAS on the plasma membrane can be inhibited and the signal transduction of carcinogenic RAS can be blocked.^137^ However, some studies showed that the degree of dependence of KRAS on PDEδ is not yet clear. For example, PDEδ knockout mice are fertile,^138^ whereas the knockout of KRAS in mice is embryonic lethal,^139^ indicating KRAS is functional in the absence of PDEδ. Although the relationship between KRAS and PDEδ is vague, blocking KRAS membrane association is a good direction to inhibit the KRAS activity.^140^

There are many small molecule compounds that inhibit the interaction between PDEδ and KRAS.^137,141–146^ In 2018, Chen et al.^147^ discovered the novel KRAS/PDEδ inhibitors through fragment-based drug design. By applying molecular docking, they found the compound 4 and compound 5 exhibited inhibitory effects on PDEδ and KRAS interaction when the two molecules exist in a specific way (Fig. 7b). The molecular docking model showed that the distance between the benzene ring of the compound 5 and the nitrogen atom of the amide of the compound 4 was 5.3 Å; the distance between the benzene ring of the compound 4 and the nitrogen atom of the imidazole of the compound 5 was 5.0 Å. The above distances are both suitable for using an ether linker to connect the two methylene groups. Therefore, the two series of compound 6 and 7 were obtained (Fig. 7b). A further optimization of the compound 6 and 7’s structure led to the synthesis of compound 8. Compound 8 exhibited a good affinity for PDEδ (*K*_d_ = 38 ± 17 nM) (Fig. 7b). The molecular docking data showed the cyclopropyl group in compound 8 forms a hydrophobic interaction with the amino residues Ile129, Val145, and Leu147 in the PDEδ. Compound 8 also exhibited inhibitory effects in Capan-1 cells (IC_50_ = 8.8 ± 2.4 μM). The RAS family regulates MAPK and PI3K-Akt-mTOR signaling pathways, and studies showed that the compound 5 downregulates the phosphorylation levels of Akt and Erk. In sum, compound 8 induces apoptosis in Capan-1 cells.

### Inhibitors of CD40/CD40L interaction (small molecules)

T cells have an important role in the immune system. Their activation requires not only the direct stimulation of foreign antigens, but also the co-stimulus signal transmitted by the interaction of surface molecules.^148^ The CD40/CD40L pathway is one of the most important co-stimulating pathways in T-cell activation. Because of its critical role in the T-cell activation, the aberrant CD40/CD40L pathway is responsible for various pathological conditions. CD40 is a membrane surface molecule has a key role in B-cell development and activation. It is a surface antigen associated with T cells and B cells function.^149^ CD40L, a T-cell–B-cell-activating molecule, is widely expressed in the activated T cells, especially CD4^+^T cells.^150^

**Fig. 8:**
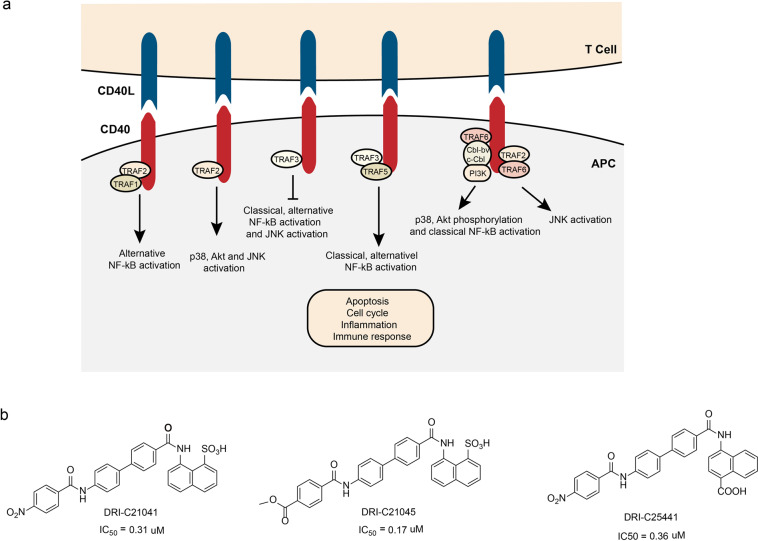
The CD40/CD40L interactions and inhibitors. **a** CD40/CD40L signal transduction and cellular response. After interacting with CD40L, CD40 recruits and interacts with tumor necrosis factor receptor-associated factor (TRAF) proteins. The activation of CD40-CD40L axis results in cellular events. **b** The chemical structures of inhibitors of CD40/CD40L

The CD40 and CD40L are a pair of complementary protein molecules. The CD40 is a member of the tumor necrosis factor receptor superfamily and its ligand CD40L (also known as CD154) belongs to the tumor necrosis factor family.^151^ Both CD40 and CD40L are mainly expressed by T and B cells. As a pair of the membrane proteins, CD40/CD40L participates in various vital physiological processes including B-cell activation, proliferation, differentiation, antibody production, apoptosis, T-cell activation, cytokine production, humoral immunity, cellular immunity, and inflammatory response^152^ (Fig. 8a). The abnormal expression of CD40/CD40L is closely related to the occurrence and development of inflammatory reaction, autoimmune diseases and immunodeficiency diseases.^152–155^ Therefore, blocking the interaction between CD40 and CD40L may has great potential to treat the associated diseases.

Multiple antibodies that block the interaction of CD40 and CD40L have been tested in preclinical or clinical trials, including: bleselumab, lucatumumab, and dacetuzumab, etc.^156^ Dacetuzumab is a IgG1 humanized anti-CD40 monoclonal antibody. In the absence of IL-4 and CD40L, dacetuzumab activates the B cells’ proliferation but inhibits the highly differentiated B cells’ proliferation. Besides, dacetuzumab transmits apoptosis signals through caspase-3, mediates antibody-dependent cell-mediated cytotoxicity (ADCC) and antibody-dependent cellular phagocytosis (ADCP) effects.^157^ However, most of these antibodies’ trails were terminated due to the severe thrombolysis side effect.^158–160^ Previous studies demonstrated the thrombolytic side effect may be a feature of antibody treatment.^158^ What is more, recent studies discovered that the antibody aggregation induced by the mAb Fc domain is also associated with thrombolysis side effect.^161^ Therefore, to avoid the thrombolysis severe side effect, alternative approaches like using small molecule compounds to block the interaction between CD40 and CD40L need to be developed. Buchwald’s group reported small molecular organic dyes that blocked the interaction between CD40 and CD40L.^162,163^ Based on these organic dye compounds, they synthesized a series of small molecule compounds that block the interaction between CD40 and CD40L.^164^ Among them, the IC_50_ of DRI-C21041, DRI-C21045, and DRI-C25441 were 0.31, 0.17, and 0.36 μM, respectively (Fig. 8b). These compounds also showed inhibitory effects on CD40L-induced B-cell activation, proliferation, and the activation of NF-κB. In addition, they also inhibit the immune response induced by alloantigen.

### Inhibitors of Skp2/Skp1 interaction (small molecules)

**Fig. 9:**
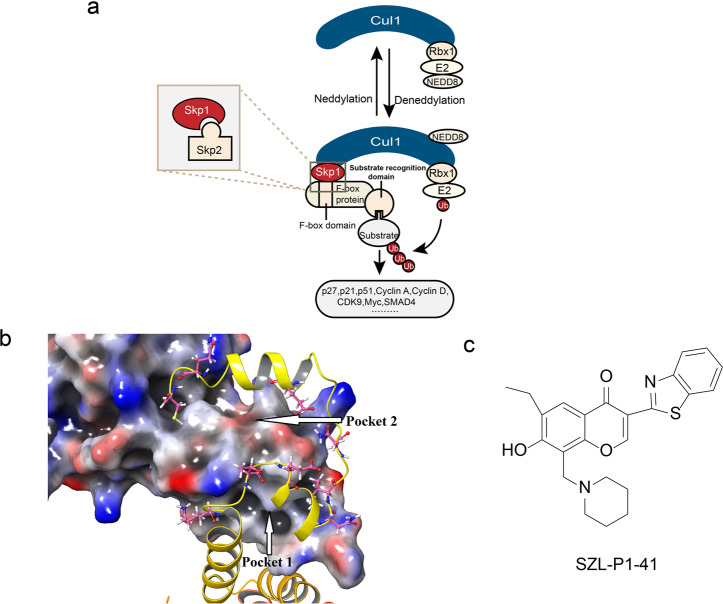
The Skp2/Skp1 interactions and inhibitors. **a** The composition of Skp2–SCF complex. Cullin 1 (Cul1) forms the backbones of ubiquitin ligase complexes. Cul1 is activated by covalent conjugation with NEDD8. The SCF complex consists of the invariable components Rbx1 (RING-finger protein), Cul1 (scaffold protein), and Skp1 (adaptor protein) as well as a variable F-box-protein component, which is responsible for substrate recognition. Skp2 is a member of the F-box protein and is a substrate recognition subunit of the SCF complex. Skp2 can specifically recognize the substrate and mediate its ubiquitination degradation. **b** The potential-binding pockets on the interface of Skp2–Skp1 complex (PDB:1FQV). **c** The chemical structures of inhibitors of Skp2/Skp1

Ubiquitin-protein degradation system (UPS) is composed of more than 1000 proteins. As the main pathway of protein degradation in cells, UPS has a key role in cell cycle regulation, intracellular signal transduction, gene transcription, metabolic regulation, immune surveillance and other basic cell life processes. The aberrant UPS system is responsible for the occurrence of various diseases.^165,166^ UPS consists of ubiquitin-activating enzyme 1 (E1), ubiquitin-conjugating enzyme 2 (E2), ubiquitin-protein ligase 3 (E3) and proteasome.^167^ At present, the E3 has been studied most. Skp1-Cullin 1-F-box (SCF) ubiquitin ligase containing F-box protein is one of the most important ubiquitin ligases and has attracted wide attention.^168^ SCF is a multi-subunit structure consisting of four parts: Cull, Skp1, Rbx1 and F-box^169^ (Fig. 9a). As a member of the F-box protein family, S phase kinase-associated protein 2 (Skp2) and Skp1, Cull, and Rbxl constitute E3 ligase, which is involved in the process of catalyzing the transformation of cells from G1 to S phase.^170^ The overexpression of Skp2 is extremely common in human cancer cells, and Skp2 overexpression promotes cancer invasion and metastasis.^171^ The interaction between Skp2 and Skp1 is the precondition of the completeness of Skp2–SCF complex and the key to exerting its E3 ligase activity. Therefore, blocking the interaction between Skp2 and Skp1 prevents the formation of Skp2–SCF complex and thus may inhibit the occurrence and development of tumors.

The crystal structure of the Skp2–SCF complex shows that Skp2 interacts directly with Skp1 through its F-box domain and indirectly binds to Cul1 and Rbx1.^172^ Along with the Skp2–Skp1 interface, Chan et al. also reported that Skp2 has 19 “hot-spots” amino acids in contact with Skp1. They classified these key Skp2–Skp1-binding sites into two pocket regions.^173^ The first region (pocket 1) is near the N terminal of Skp2 and is in the F-box motif which including the amino acid residues of Trp97, Phe109, Glu116, Lys119, and Trp127. The second region (pocket 2) is close to the C terminal of Skp2, formed by a Leu-rich repeat sequence with some residues from the F-box domain (Fig. 9b). Inhibitors bind to one or both of these pockets prevent the formation of Skp2–Skp1 complex.

Chan et al.^173^ identified seven compounds that could inhibit the formation of Skp2–Skp1 complex through HTS. Among which, SZL-P1-41 exhibits strong inhibition effects to the Skp2–Skp1 complex formation (Fig. 9c). The molecular docking model shows the SZL-P1-41 binds to pocket 1 rather than pocket 2, which suggests the pocket 1 in the F-box sequence of Skp2 may have a leading role in the Skp2–Skp1 interaction^173^ (Fig. 9b). The docking model also suggests that the benzothiazole structure of SZL-P1-41 interacts with the Trp97 residue on Skp2 through an aromatic stack and a polar contact; The flavone groups of SZL-P1-41 interact with the Asp98 and Trp127 of Skp2 via hydrogen bonding or hydrophobic interaction; The ethyl group on phenol ring extends into Skp1 region; The piperidine interacts with both Asp98 and Trp127. Both in vitro and in vivo experiments results showed the Skp2 inhibitors could inhibit the Skp2-mediated P27 ubiquitination. The in vivo experiments data also showed the SZL-P1-41 could effectively inhibit the growth of tumors. In addition, the Skp2 inhibitors not only inhibit the formation of Skp2–Skp1 complex, but also reduce the Skp2 E3 ligase activity. The higher doses of SZL-P1-41 also reduces the Skp2 protein expression.

### Inhibitors of Keap1/Nrf2 interaction (small molecules, peptides)

**Fig. 10:**
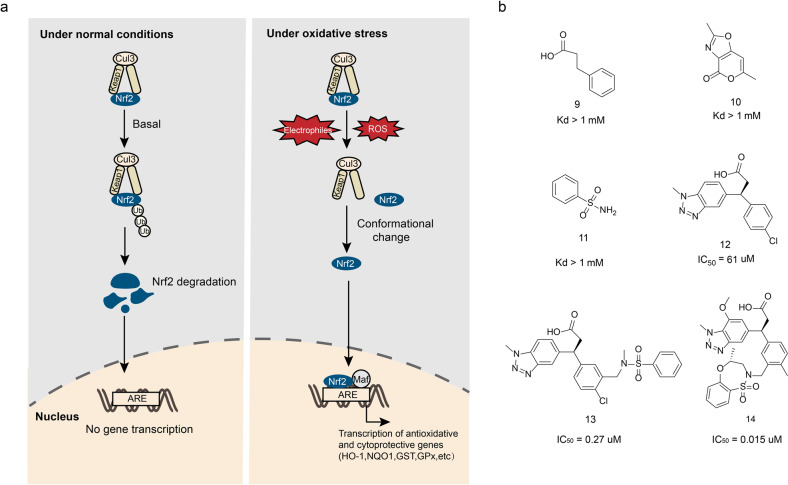
The Keap1/Nrf2 interactions and interactions. **a** The Keap1–Nrf2-ARE pathway. Under basal conditions, Nrf2 binds to Keap1 and is degraded by proteasomes. Under oxidative stress, Nrf2 escapes the degradation mediated by Keap1 and transfers to nucleus, binding with ARE and Maf protein to initiate the transcription of antioxidative and cytoprotective genes. **b** The chemical structures of inhibitors of Keap1/Nrf2

The Keap1–Nrf2-ARE signaling pathway is the most important antioxidant stress pathway, which is associated with a variety of oxidative stress-related diseases including cancer, Alzheimer’s disease, Parkinson’s disease, diabetes, and arthritis.^174^ Under physiological conditions, the Keap1 targets the Nrf2 to initiate the ubiquitin-dependent degradation of protein media. When cells under electrophilic or oxidative stress, the Nrf2 escapes the Keap1-mediated degradation and enters the nucleus, where it mediates the activation of the antioxidant and cytoprotective genes^175,176^ (Fig. 10a). Therefore, the Nrf2 signaling pathway activators should have therapeutic effect in oxidative stress-induced diseases. Up to date, most of the “Nrf2 activators” are inhibitors of Keap1/Nrf2 interaction which covalently bind with the sulfhydryl groups on the cysteine in Keap1 through oxidation or alkylation. The covalent adduct changes the Keap1 conformation that prevents the Nrf2 interact with Keap1.^177^ However, the covalent binding is irreversible. As a result, the long term application of the Keap1/Nrf2 inhibitors results in the accumulation of the active Nrf2, which may trigger other problems like cancer^178^ Therefore, finding non-covalent small molecules that directly interfere with the Keap–Nrf2 interaction, dissociating the two and exerting antioxidant defense effects has become a new therapeutic strategy.^179^

In 2006, Hannink’s group analyzed the structure of a complex between the Kelch domain of Keap1 and Nrf2-derrived peptide, thus revealed the binding interface between Nrf2 and Keap1, and determined the key residues of Keap1, including Arg380, Arg415, Arg483, Ser363, Ser508, Ser555, and Ser602, which laid the foundation for the design of Keap1/Nrf2 interaction inhibitors.^180^

The study of the Keap1/Nrf2 inhibitors begins with the investigation of the polypeptides that inhibits the Keap1/Nrf2 interaction. Up to date, a number of the inhibitive polypeptides have been reported^181–184^ (Table 4). Hu et al.^185^ developed a series of fluorescent probes to verify when the peptide chain length is 9 amino acids it has the best activity to inhibit the Keap1/Nrf2 interaction. The peptide inhibitor P1 designed based on the fluorescent probes has a moderate inhibitory activity (IC_50_ = 3480 ± 920 nM), and its activity increases with the elongation of the polypeptide chain (7–16 amino acids). For instance, the hexadecapeptide P2 (IC_50_ = 163 ± 11 nM) exhibits the highest activity.^181^ The acetylation of the N terminus of such a peptide neutralizes the positively charged group at the N terminus, which also greatly changes its electrical property. The nonapeptide P3 was obtained via the modifications as above-mentioned and exhibits great activity (IC_50_ = 194 ± 49 nM).^186^ Subsequently, the structure–activity relationship study demonstrated that the heptapeptide also shows activity, such as heptapeptide P4 (IC_50_ = 8230 ± 262 nM) and P5 (IC_50_ = 558 ± 53 nM), which exhibits moderate inhibitory activity. The follow-up work focused on acetylation and C18 fatty acid stearic heptapeptide. The compound P6 showed excellent Keap1 inhibitory activity (IC_50_ = 22 ± 3 nM).^182^ However, the peptide inhibitor has large molecular weight and poor ability to penetrate the cell membrane. Therefore, it is of great significance to find a class of small size peptide inhibitors with strong membrane permeability. Steel et al. designed and synthesized a number of highly permeable membrane peptides. Among them, the compound P7 induces the expression of heme oxygenase-1 (HO-1) in cells, and inhibits the proinflammatory cytokine-TNF’s expression.^187^

**Table 4:** Some peptide inhibitors of Keap1/Nrf2 interaction

Name	Sequence	IC_50_ (nM)
P1	H-LDEETGEFL-OH	3480 ± 920
P2	H-AFFAQLQLDEETGEFL-OH	163 ± 11
P3	Ac-LDEETGEFL-OH	194 ± 49
P4	Ac-NAETGEF-OH	8230 ± 262
P5	Ac-DAETGEW-OH	558 ± 53
P6	St-DPETGEL-OH	22 ± 3
P7	YGRKKRRQRRRLQLDEETGEFLPIQ	24

Most of the high binding peptides have poor cell permeability. Subsequently, the high binding peptides’ activity is not ideal. Therefore, screening the small molecule inhibitors have become a hot spot in the study of Keap1/Nrf2 interaction inhibition.^188–195^ The mile stone of the small molecule inhibitors study is the development of benzothiazepine heterocyclic Keap1–Nrf2 small molecule inhibitors made by GSK through fragment-based drug design.^189^ After screening 330 fragments via X-ray crystallography, the compounds 9–11 (Fig. 10b) were identified which interact with Arg483, Tyr525, and Ser602, respectively. The binding of the compounds to the amino acids as mentioned above simulating the binding of Nrf2 peptide segment with Keap1, but the binding activity of these three compounds was low (*K*_d_ > 1 mM). To improve the compounds’ Keap1-binding activity, various structural modifications were made. Among which, the introduction of methanesulfonamide on the benzene ring of compound 12 significantly increased the compound’s Keap1-binding activity by 20 times (IC_50_ = 61 μM). When compound 13 was completely introduced, its IC_50_ strikingly reached 0.27 μM. A series of structural optimizations were performed using compound 13 (Fig. 10b) as the lead: when methyl group is substituted for the chlorine atom on the benzene ring, it can release its potential binding to the sulfonamide center; Introducing an electron-donating group methoxy group at the 7-position of benzotriazole can improve hydrogen bonding and enhance surface bonding; The conversion of benzenesulfonamide ring to seven membered benzothiazide heterocycle can make more space occupied by sulfonamide and benzotriazole sites, and the activity of compound 14 (Fig. 10b) is significantly increased (IC_50_ = 0.015 μM). These compounds can induce the expression of Nrf2 downstream target protein NQ01 in BEAS-2B cells, and reduce the lung inflammation induced by ozone in animal experiments.

### Inhibitors of PD-1/PD-L1 interaction (small molecules, peptides, antibodies)

**Fig. 11:**
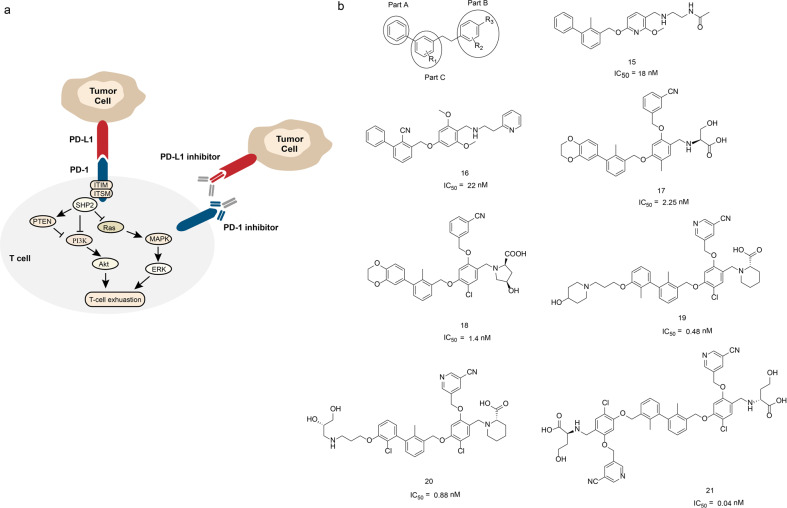
The PD-1/PD-L1 interactions and inhibitors. **a** PD-1/PD-L1 signaling pathway. PD-1/PD-L1 interaction causes the phosphorylation of ITIMs and ITSMs in the intracellular domain of PD-1, and then recruits SHP2 to suppress PI3K/Akt, Ras/MAPK/ERK signaling pathway, leading to T-cell exhaustion. **b** The chemical structures of inhibitors of PD-1/PD-L1

Studies indicate that the PD-1/PD-L1 signaling pathway has critical role in tumor immune escape and tumor development.^196^ PD-1 (also known as CD279) is an immunosuppressive receptor belongs to the CD28 superfamily of T-cell regulatory receptors and its natural ligand is PD-L1. Under physiological conditions, PD-1 is mainly expressed in activated immune cells, which promotes the maturation of T lymphocytes, regulates unnecessary or excessive immune response through negative regulation, and maintains immune tolerance. The over activation of PD-1/PD-L1 signaling pathway negatively regulates the function of T cells, which cancels the immune system surveillance function and promotes the escape of tumor cells.^197^ Therefore, blocking the interaction of PD-1 and PD-L1 maintain the T cells immune function may be a potential strategy for tumor treatment (Fig. 11a). The PD-1/PD-L1 signaling pathway inhibitors include monoclonal antibodies, peptides, and small molecule inhibitors.

Up to date, there are five monoclonal antibody drugs Pembrolizumab (Keytruda), Opdivo (Nivolumab), Tecentriq (Atezolizumab), Bavencio (Avelumab), and Imfinzi (Durvalumab) have been approved as PD-1/PD-L1 signal pathway inhibitors for the treatment of melanoma, non-small cell lung cancer and other diseases^198–202^ (Table 5). Pembrolizumab is the first PD-1 inhibitor approved by the FDA for the treatment of advanced or unresectable melanoma, which does not respond to other drugs.^203^ Pembrolizumab is a highly selective humanized IgG4-κ anti-PD-1 monoclonal antibody, which activates tumor infiltrating lymphocytes (TIL). The combination of PD-1 highly expressed in TIL and PD-L1 expressed in tumor cells is an important factor for tumor immune escape. Pembrolizumab binds to PD-1 on the surface of TIL and inhibits its interaction with PD-L1/2 to activate TIL. Although immunotherapy against PD-1/PD-L1 has been applied in clinic, the use of monoclonal antibodies may affect the proliferation and activation of T cells, thereby trigger severe immune-related adverse reactions, including tissues damage, weaken the function of Fc-immune effect (killing immune cells), etc.^204,205^

**Table 5:** Antibody agents targeting PD-1/PD-L1 approved by FDA

Name	Diseases	Developer
Keytruda	Melanoma, non-small cell lung cancer	Merck Sharp & Dohme
Opdivo	Melanoma, head and neck cancer	Bristol Myers Squibb
Tecentriq	Non-small cell lung cancer, bladder cancer	Roche
Bavencio	Merkel cell carcinoma	Merck and Pfizer
Imfinzi	Urothelial carcinoma	AstraZeneca

Compared with monoclonal antibodies, the peptides and small molecule drugs do not have the limitations of monoclonal antibodies as mentioned above.^206^ Chang et al.^207^ developed the first hydrolysis-resistant d-peptide antagonists to target the PD-1/PD-L1 pathway by using the mirror-image phage display (Table 6). The optimized compound ^D^PPA-1 binds to PD-L1 at an affinity of 0.51 μM in vitro. The cellular level blockade assay data and tumor-bearing mice experiments results all indicate that the ^D^PPA-1 disrupts the PD-1/PD-L1 interaction under in vivo condition.^207^ Aurigene developed a small peptide AUNP-12, which is an anti-PD-1 targeted immunotherapy for cancer (the structure of the compound has not been disclosed).^208^ The AUNP-12 inhibits the binding of PD-1 and PD-L1 under in vitro conditions (IC_50_ = 0.72 nM), but the time of drug metabolism was short. The animal trials data demonstrated the AUNP-12 has good anti-PD-L1 activity and effectively inhibits the growth and metastasis of tumor cells.

**Table 6:** Peptide inhibitors of PD-1/PD-L1 interaction reported by Chang et al.

Name	Sequence	*K*_d_ (μM)
^D^PPA-1	NYSKPTDRQYHF	0.51
^D^PPA-2	KHAHHTHNLRLP	1.13
^D^PPA-3	AAKMDGHLHGGQ	No binding
^D^PPA-4	TLYQRPSTNLER	22.0
^D^PPA-5	RHTNDYSQFYPK	No binding

Because the structure of PD-1 and PD-L1 proteins are not available, the development of small molecule PD-1/PD-L1 inhibitors lags far behind the development of antibody drugs. By analyzing the PD-1/PD-L1 complex structure, Zak et al.^209^ reported that there are three main binding pockets in the contact interface between PD-1 and PD-L1, which provide a rational basis for drug development. With the success of PD-1 monoclonal antibodies and macromolecular biomedical drugs, Bristol Myers Squibb (BMS) conducted an in-depth investigation of small molecule inhibitors of the PD-1/PD-L1 pathway. In 2015, the company disclosed its first patent on a biphenyl immunomodulator. The homogeneous time-resolved fluorescence (HTRF) test results demonstrated that these compounds blocked the interaction between PD-1 and PD-L1. Surprisingly, some of the compounds even reached nanomolar activity. The IC_50_ of representative compounds 15 and 16 (Fig. 11b) were 18 and 22 nM, respectively.^210^ In the same year, in another patent disclosed by BMS, additional structural modifications have been made which include the benzene ring in part A of the compound was replaced by 1,4-benzodioxane, and m-cyanobenzene was introduced into the benzene ring of part C through an ether bond. The structural optimization as above-mentioned significantly improved the compound’s PD-1/PD-L1 inhibition activity that their IC_50_ values reached 0.6–10 nM range. Specifically, the IC_50_ of representative compounds 17 and 18 (Fig. 11b) were 2.25 and 1.4 nM, respectively.^211^ To improve the compound’s inhibition activity further, the researchers continued to optimize the structures of this class of compounds. The additional structural modifications include introducing different hydrophilic groups into a part of the hydrophobic biphenyls through a carbon chain, which improved the compounds’ activity further. The representative compounds 19 and 20’s IC_50_ values were 0.48 and 0.88 nM (Fig. 11b), respectively.^212^ In 2018, BMS disclosed new compounds with symmetric structures. Compare to other compounds, the new compounds replaced the original groups on the left side with new groups that have the same or similar structures as the ones on the right side based on the original structure, thus forming a compound characterized by “central symmetry”. The activity of this type of compounds is generally less than 1 nM, and the representative compound 21’s IC_50_ values (Fig. 11b) reaches 0.04 nM.^213^

## Stabilizers of PPIS

### Stabilizers of 14-3-3/H^+^-ATPase (small molecules)

The tyrosine 3-monooxygenase/tryptophan 5-monooxygenase activator protein family (14-3-3 protein) has an important role in PPIs. It is a highly conserved ubiquitous protein family encoded by multiple genes in most organisms.^214^ There are at least seven highly conserved subtypes of 14-3-3 proteins encoded by different genes in mammals. The 14-3-3 proteins bind to various ligand proteins including kinases, phosphatases, and transmembrane receptors.^215^ The 14-3-3 proteins regulate more than 500 endogenous molecules’ activity through binding to them.^216^ Since these endogenous molecules have critical roles in the cell metabolism process, cell cycle modulation, apoptosis, cell differentiation, transcription, signal transduction, and other vital biological events, the intervene of these molecules’ activities may yield severe consequences in the cells.^217,218^ Specifically, the 14-3-3 proteins also named as “bridge protein” of protein–protein interactions as they bind with transcription factors to form complexes that regulate the expression of associated genes. Due to the important function of 14-3-3 proteins in cells, they have critical roles in various diseases including the nervous system diseases, arthritis, malignant tumors, and infectious diseases etc.^218–220^

All 14-3-3 proteins have similar tertiary structures and the structure can be divided into three parts: N terminal, conservative core region, and C terminal. Each monomer consists of 9 helium spirals (ɑA~ɑI) located between the N terminal and the C terminal that arranged from an anti-parallel to an L-shaped structure separated by a short loop.^221^ Under certain conditions, the 14-3-3 proteins can aggregate together in the form of stable homologous/heterodimers that can bind with two ligands simultaneously.^222^ The dimer formation is a necessary regulatory step for its binding to the ligand protein. The dimer interface consists of αA from one monomer with the combination of and αC and αD from another monomer forming highly conserved facultative grooves. The grooves contain both polar and nonpolar amino acid residues and also contain an intense negative charge. The nonpolar amino acid residues in all subtypes of 14-3-3 proteins mainly distribute along the inner grooves, while the polar amino acid residues are located on the outer surface of the grooves. Such a unique distribution of nonpolar-polar amino acid residues makes the grooves identify the target proteins with common characteristics.

**Fig. 12:**
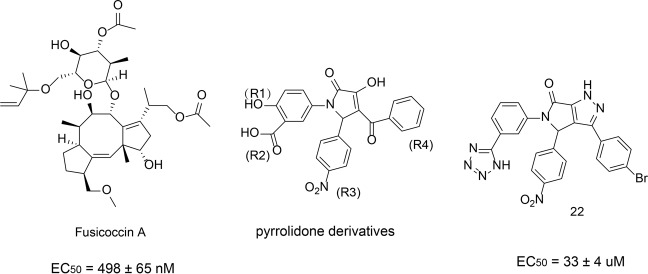
The chemical structures of stabilizers of 14-3-3

Fusicoccin A, a natural product, is the first reported stabilizer to regulate the interaction between the 14-3-3 protein and its ligand (Fig. 12). Fusicoccin A is a diterpenoid glycoside with a 5-8-5 ring that binds to 14-3-3 receptors. Fusicoccin A stabilizes the complex formed by 14-3-3 protein and plasma membrane ATPase (PMA).^223^ The crystal structure studies showed that the 14-3-3 dimer forms a complex structure with 52 amino acids at the C terminal of H^+^-ATPase, and Fusicoccin A fills the gap between the protein–protein interface of the complex.^223^ The hydrophobic 5-8-5 ring is inserted into the binding channel of the 14-3-3 protein. The bottom of the hydrophobic cavity contains Val153, Phe126, and Met130. The methyl or methoxy substituent is an important condition for contacting the hydrophobic bottom. The 5-8-5 ring has extensive hydrophobic interactions with Pro174, Ile174, Gly178, Leu225, Ile226, and Ile956 of H^+^-ATPase proteins. In addition, many water-mediated polar interactions were formed between Fusicoccin A and 14-3-3 proteins.

Richter et al.^224^ reported that the pyrrolidone derivatives could stabilize the interaction between 14-3-3 and PMA. Among these, the compound 22 exhibited the highest activity (Fig. 12). The crystal structure of the pyrazole derivatives and 14-3-3/PMA complex showed that the rigid pyrazole part penetrated into the protein–protein interaction interface deeply, therefore enlarged the interface with PMA. Compared with the natural product Fusicoccin A (EC_50_ = 498 ± 65 nM), the activity of compound 22 was better (EC_50_ = 33 ± 4 μM). Furthermore, compound 22 showed a good selectivity and has no effect on 14-3-3/C-Raf or 14-3-3/p53 interactions.

### Stabilizers of S100 pentamer (small molecules)

**Fig. 13:**
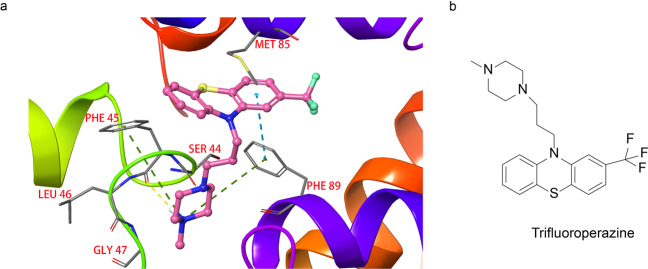
Proteins and small molecule inhibitors of S100 pentamer. **a** The binding modes of trifluoroperazine binds to S100 (PDB:3KO0). Due to the clarity, only two adjacent S100A4 monomers and their contact interface are shown. **b** The chemical structure of a stabilizer of S100 pentamer

The S100 protein was given its name because it is well soluble in 100% saturated ammonium sulfate under neutral conditions.^225^ Up to date, at least 20 members of the S100 protein family have been identified, including S100A1-A15, S100B, S100P, etc.^226^ The S100 proteins mainly exist in the forms of homodimers, heterodimers, trimers, and tetramers in the cells.^227^ Previous studies have shown that the S100 proteins act as a calcium sensor, which regulates many intracellular and extracellular activities in a calcium-dependent manner.^228^ The binding of calcium ions changes the S100 protein conformation, exposing its binding sites to the target proteins. Therefore, various biological functions of S100 protein can be exerted through regulating the calcium ions under in vivo conditions.^229^ For example, S100 regulates protein phosphorylation, enzyme activity, cell proliferation, cell differentiation, inflammatory reaction induction, and protects cells from oxidative damage.^225,229^ Studies showed that the high expression of S100A4 associate with rheumatoid arthritis, kidney fibrosis, and cardiac hypertrophy.^230,231^ Garrett et al.^232^ reported several phenothiazines that block the activity of S100A4. One of these compounds, trifluoroperazine inhibits the S100A4 function through stabilizing its inactive pentamer (Fig. 13b). The complex structure study discovered that the trifluoroperazine forms a pentamer complex with S100A4 and the two molecules are in contact with each other at the interface. Further analysis of the complex structure found that trifluoroperazine binds to a hydrophobic patch, which includes the side chains of Ile82, Met85, and Cys86, from one protomer and Phe89 as well as Phe93 from the other (Fig. 13a). The methylated piperazine ring of trifluoroperazine interacts with the protomer of Ser44, Phe45, Leu46, and Gly47. In addition, the carbonyl oxygen atom of the protomer Phe45 forms a hydrogen bond with the nitrogen atom on the piperazine ring.

### Stabilizers of influenza nucleoprotein protomers (small molecules)

Influenza virus is the pathogen causing acute infectious disease influenza. Influenza virus nucleoprotein (NP) is its main structural protein and the main component of nucleocapsid.^233^ The ribonucleoprotein complex is composed of ribonucleoprotein and RNA fragments of virus and three kinds of dependent RNA polymerase PA, PB1, and PB2, which participate in the transcription, replication, and assembly of the virus. As the main structural protein of the virus, nucleoprotein contains many functional domains, such as nuclear localization sequences, RNA-binding domains, NP–NP-binding domains, and PB2-binding domains. All these domains have vital functions that are indispensable components of viral replication. Therefore, inhibiting the nucleoprotein function may have antiviral effects.

**Fig. 14:**
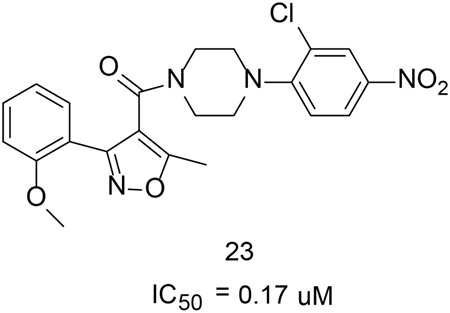
The chemical structures of stabilizers of influenza nucleoprotein

Gerritz et al.^234^ reported a triazole compound that induces the formation of higher-order nuclear protein oligomers, which prevents the nuclear proteins entering the nucleus, thereby inhibiting viral replication. Previous studies showed that the binding sites of the triazole compound might be located in two regions on NP: one in NPY289/N309 region and the other in NPY52 region. Six molecules of compound 23 (Fig. 14) bridge two NP trimers (NP_A, NP_A′, NP_A″ and NP_B, NP_B′, NP_B″) to form a hexamer. The structure analysis on compound 10 and NP protein complex showed that compound 10 located between the interfaces of two trimers and stabilizes the complex.^234^ The other unique structures of the triazole compounds and NP complex include a hydrophobic pocket formed between two NP monomers by the amino acid residues Tyr289, Phe291, Try296, Tyr52, and Tyr313 on each monomer. The nitro moiety on the aromatic ring of compound 23 forms a Π-Π interaction with Tyr289 on NP_A. The piperazine moiety of compound 23 forms a hydrophobic interaction with Tyr254 on NP_B. Further, the hydroxyl group of the NP_B Ser forms a hydrogen bond with the carbonyl group of compound 23.

### Stabilizers of microtubules (small molecules)

The microtubule is the main component of the cytoskeleton, which is composed by α-tubulin and β-tubulin. Microtubules have a vital role in maintaining cell morphology, cell division, signal transmission, and material transport.^235^ In the living cells, microtubules aggregate with each other into spindles in the early stages of cell division. The spindles pull chromosomes to move towards the two poles into two daughter cells during mitosis, thereby completing cell proliferation. Under physiological conditions, there is a dynamic balance between the microtubule and tubulin dimer. The microtubule stabilizers stabilize microtubules and promote the multimerization of microtubules, thus block the depolymerization of microtubules, and thereby destroy the dynamic instability of tubulin. Such effect further destroys the rapidly differentiated tumor cells during mitosis, stagnates the cell cycle, and in turn induces the tumor cells to undergo apoptosis.^236^

**Fig. 15:**
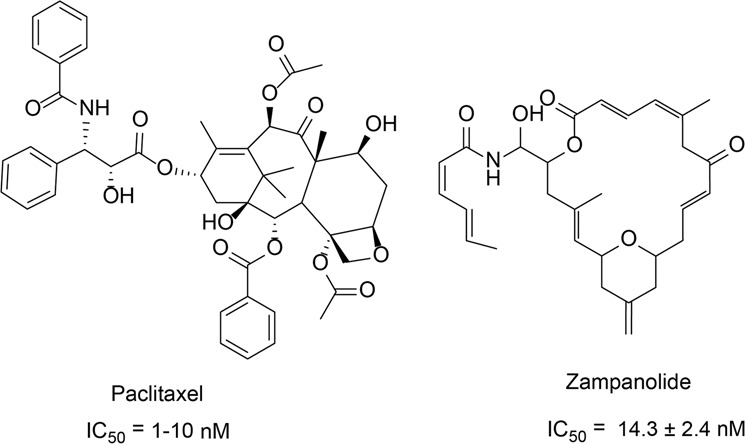
The chemical structures of stabilizers of microtubules

Paclitaxel (Fig. 15) is the first approved microtubule stabilizer. Studies showed that the Paclitaxel binds with β-tubulin, promotes the aggregation of microtubulin, stabilizes microtubule structure, hinders the formation of spindles, and leads to cell cycle arrest in G2/M phase.^237,238^ Zampanolide (Fig. 15) is a 20-membered macrolide isolated from the Tongan marine sponge Fasciospongia rimosa.^239^ It arrests cells in mitosis and inhibits cell proliferation by stabilizing microtubules. The structural analysis shows that zampanolide induces the disordered curled M-loop into an ordered spiral structure through its side chain.^240^ The M-loop is composed of eight amino acid residues in the middle region of the tubulin subunits, which maintains the interaction between the microtubule fibrils. The change in the M-loop facilitates the lateral contact between the microtubule fibrils and thus stabilize the microtubules. In 1A9 cells, zampanolide exhibited a IC_50_ value of 14.3 ± 2.4 nM, which demonstrated itself a potential microtubule stabilizer.^241^

## Conclusion

In recent years, new PPIs modulators development has been an attractive goal in preclinical studies.^242,243^ However, the design of modulators targeting PPIs still faces tremendous challenges. Besides the challenges mentioned previously like the difficult PPI interfaces for the drug design, lack of ligands reference, the ineffectiveness of the classic medicinal chemistry approaches for PPI drug development, lack of guidance rules for the PPI modulators development, the biggest obstacle is the lack of high-resolution PPI proteins structures. Because the medicinal PPI drug design is based on the high-resolution PPI protein structures, more resources should be put into the structural biological studies of the identified PPIs.

Inhibitors and stabilizers are two ways to modulate PPIs. Some of these modulators have been applied in the clinic, some have entered clinical trials, and some have lead compounds that require further structural optimization. Although compounds such as trifluoroperazine and zampanolide exhibit PPIs stabilizing activity, the PPIs stabilizers’ development has not received sufficient attention as compared to the PPIs inhibitors’ development.^63^ The difficulties for the PPIs stabilizers’ development include the insufficient understanding of PPIs mechanisms, the poor chemical space performance of PPI stabilizers in existing small molecule libraries, and the extreme diversity of the PPIs stabilizers’ molecular structures make it difficult to establish the criteria to guide the design of new PPIs stabilizers. Most of the identified PPI stabilizers are natural products, only a few of them are synthesized through the rational design method. The HTS of the natural products that have PPI stabilization activities may be the direction of finding the lead compounds of PPI stabilizers.

Compared with the traditional small molecule inhibitors, peptides exhibit higher affinity and specificity, making it easier to bind with the target proteins. However, the peptide has two major problems when used as drugs: its instability under in vivo conditions and their poor membrane permeability. Fortunately, new technologies are available now to counter the two problems. To prevent the quick degradation of the peptides after entering the body, the chemical modifications can be applied to improve the stability of the peptides. Regarding the peptides’ poor membrane permeability issue, there is a class of short peptides that have been found in recent years to have the function of penetrating biomembrane and mediate transmembrane transduction of macromolecular substances.^244^ This brings significant progress to the development of intracellular peptides.

In recent years, remarkable progress has been made in the development of antibodies that regulate PPI, especially the monoclonal antibodies regulate PD-1/PD-L1 interaction. However, due to the high research cost, the instability, and potential severe immunogenic side effects of antibodies, more and more attention has been drawn to the peptides and small molecular inhibitors, especially the small molecular inhibitors. Compared with antibodies, the classic small molecule drugs have advantages such as lower research costs, diverse preparations, oral administration, and better tumor microenvironment penetration.

Decades ago, due to the limited understanding of the PPI properties and very limited available screening techniques by time, the modulation of PPIs has been recognized as one of the most challenging tasks in drug discovery for a long period of time. However, the rapid development of structural biology and the associated methodologies have helped us to understand the PPI properties to a level we could not imagine before. Besides, the rapid development of various high-throughput screening approaches also makes the quick screening of the PPI modulators possible. As a result, great progress has been made in the development of PPI modulators lately. In summary, opportunities and challenges coexist in the discovery of modulators targeting PPIs. In the future, with the emergence of new and better approaches to reveal the structures of protein complexes and the development of structural biology, it is believed that more PPI small molecule modulators will be developed and enter the clinic to benefit the patients.
